# Disturbance observer-based adaptive position control for a cutterhead anti-torque system

**DOI:** 10.1371/journal.pone.0268897

**Published:** 2022-05-24

**Authors:** Hangjun Zhang, Jinhui Fang, Huan Yu, Huibin Hu, Yuzhu Yang

**Affiliations:** 1 State Key Laboratory of Fluid Power and Mechatronic Systems, Zhejiang University, Hangzhou, China; 2 Aerospace System Engineering Shanghai, Shanghai, China; Beijing University of Posts and Telecommunications, CHINA

## Abstract

To conveniently replace worn cutterhead tools in complicated strata, a novel cutterhead attitude control mechanism was recently designed. Meanwhile, the mechanism also causes an engineering problem of how to control a matching cutterhead anti-torque system (CATS) effectively, which is used to prevent a drive box of the cutterhead from rotation during a complex excavation process. In this paper, a disturbance observer-based adaptive position controller is proposed for the CATS. The proposed method presents a nonlinear adaptive controller with adaptation laws to compensate for the unknown time-varying load torque and damping uncertainty in the system. Based on the disturbance observer method and sliding mode control, an asymptotically stable controller proven by Lyapunov theory is constructed using the back-stepping technique. In addition, a virtual test rig based on MATLAB and AMESim co-simulation is built to verify the validity of the proposed controller. The simulation results show that the proposed method has good performance for tracking tasks in the presence of uncertainties compared with PID control. Together, the data support targeting disturbance observer-based adaptive position control as a potential control strategy for cutterhead anti-torque systems.

## 1. Introduction

**Fig 1:**
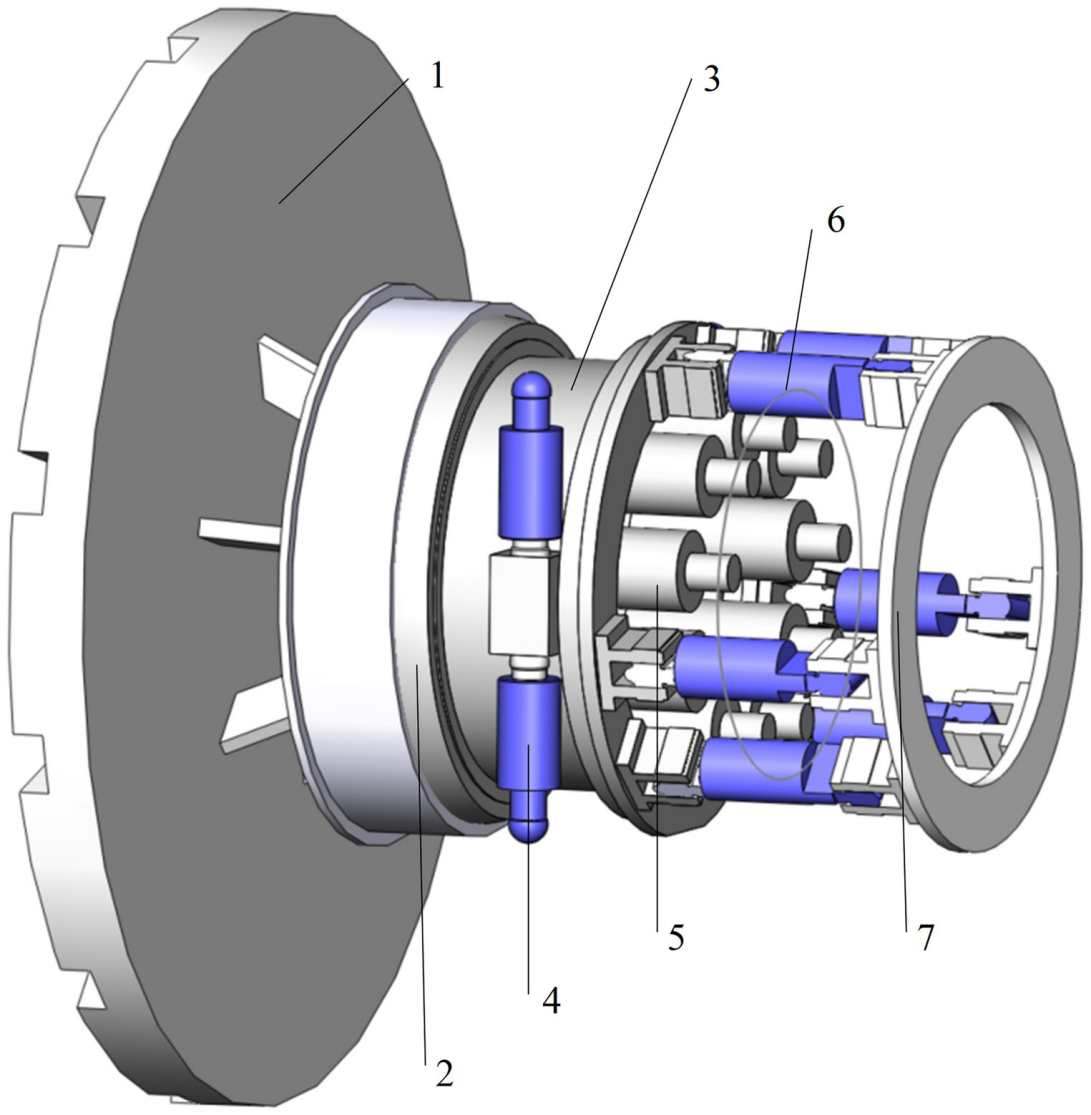
Cutterhead anti-torque system mechanism schematic. 1-Cutterhead, 2-Radial joint bearing, 3-Drive box, 4-Torque cylinder, 5-Motor, 6-Posture cylinder, 7-Support seat.

As an important carrier of underground transportation networks, tunnels have significant benefits in expanding urban space, relieving traffic congestion and exploiting natural resources. Tunnel boring machines are widely applied in tunnel construction since the shield tunnel method has distinct advantages over other methods in terms of efficiency, safety, environmental protection, etc. Among them, slurry shields are the preferred equipment for tunnel construction in high water pressure and high permeability sand strata [1–3]. To satisfy the growth of traffic demand, slurry shields have a trend toward upsizing, relying on the progress of shield construction technology. Meanwhile, large-diameter slurry shields face more challenges than normal shields in the construction process [4–7], such as excessive wear of the cutterhead and edge scrapers, instability of the tunnel face, complex attitude control of the heavy cutterhead and difficult tunnel excavation in the upper-soft and lower-hard stratum. To address these problems, tunnel boring machine research institutions and manufacturers, including SKLOFP, Herrenknecht AG, CCCC and CRCHI, have successively designed and produced a new type of cutterhead attitude control mechanism [8–10], as shown in Fig 1. The cutterhead is connected to a drive box and driven by several inner electric or hydraulic motors. The drive box is fixed on an inner ring of a radial joint bearing whose outer ring is connected to a shield mechanism. A number of circular-distributed posture cylinders are installed on the backboard of the drive box, and the other sides are connected to a support seat of the shield mechanism. If these posture cylinders are extended or retracted synchronously, the cutterhead will move forward or backward. Therefore, an over-excavating specific soil space is constructed, which is used to replace the cutterhead worn tools or get out of shield jamming. The posture cylinders are different from the thrust cylinders used for forward digging in the whole excavation process. In addition, the inner motors drive the cutterhead to rotate through gears, and the cutterhead cuts the front soil and receives a reaction torque that drives the drive box to rotate radially. Due to the floating state of the drive box, a matching cutterhead anti-torque system (CATS) is designed to transmit the reaction torque to the shield structure and avoid drive box rotation against the shield. The main feature of the CATS is that two pairs of torque cylinders are equipped on both sides of the drive box symmetrically. The force of torque cylinders acts on the shield mechanism to provide reverse support and prevent the drive box from rotation during an excavation process.

The radial attitude of the drive box is controlled in real time by these torque cylinders driven by hydraulic power. Therefore, properly controlling the torque cylinders is the key technology to keep the drive box stable and improve tunnel construction efficiency and safety. PID control is currently the most mature and widely used control method in analog control systems, and it is also used in CATS control. However, hydraulic systems exhibit strong nonlinearities, such as nonlinear valve flow-pressure characteristics and temperature-varying oil properties. Hydraulic systems also have some model uncertainties, such as damping coefficients and unknown time-varying load forces. Both drawbacks cause control difficulties, in turn [11, 12]. Conventional PID controllers cannot achieve ideal control effects, and have poor adaptability to operating conditions. To obtain better dynamic performance, an increasing number of researchers have used nonlinear control strategies to compensate for nonlinearities and uncertainties. Under nominal operating conditions, the pole placement method [13] or adaptive control [14] was used to locally linearize the nonlinear dynamics. However, these controllers cannot provide excellent performance under all working conditions. The feedback linearization method [15], based on cancelling nonlinear terms, was used without taking system uncertainties into consideration. In addition, sliding mode control [16–19] has been widely used in hydraulic control systems. The dynamic behaviors on the sliding mode surface cannot be affected by model uncertainties and disturbances. However, the inherent chattering of the control input may cause high-frequency vibration and degrade the control performance. The concept of adaptive control [20, 21] was proposed in the research of autopilots of high-performance aircraft. This controller has a weak antidisturbance ability, although it is considered a valid method to overcome model uncertainties. The deterministic robust control method [22, 23] was applied to control nonlinear systems. In references [24, 25], Yao and Tomizuka developed adaptive robust control that effectively integrates adaptive control and deterministic robust control. They [26] applied a nonlinear adaptive robust controller for trajectory tracking of hydraulic actuators with uncertain nonlinearities and model uncertainties and achieved satisfying results. Furthermore, a desired compensation adaptive robust control framework [27] was also presented for nonlinear systems with uncertain nonlinearities and model uncertainties. In addition, many researchers applied a disturbance observer [28–31] to overcome the load disturbance and model uncertainties in complex systems, which had good trajectory tracking performance. Many observers provided an asymptotic or exponential convergence implying the error convergence to zero over an infinite time interval. In order to achieve a finite-time observation, several strategies have been presented and the sliding mode observer is one of the main methods with widespread applications. But the sliding mode observer is affected by some restrictions, leading to the potentially destructive chattering phenomenon in its convergence to zero. To eliminate the shortcoming, alternative approaches have been developed, such as the high-order sliding mode observer to reduce the chattering and the terminal sliding mode observer with finite-time convergence properties [32, 33]. Currently, to obtain better control performance, various nonlinear algorithms begin to be integrated [34–37], such as combining disturbance observer and sliding mode control, disturbance observer and adaptive control. Their experimental results showed the efficiency of novel nonlinear control strategy, although its structure was complex and difficult to be constructed. Besides, Chen and Zhu proposed a novel adaptive neural network observer in which the adaptive laws were constructed using quantized measurements. And accurate estimations of state and actuator efficiency factor can be obtained despite actuator degradation [38]. Zhu also studies the observer-based output feedback control problem for a class of cyber-physical systems with periodic denial-of-service attacks. By means of a cyclic piecewise linear Lyapunov function approach, the observer-based controller design was carried out and its effectiveness was verified by unmanned ground vehicles under periodic denial-of-service attacks [39]. Slurry shields work in complicated strata, and the force with disturbance on the cutterhead is unknown and time-varying. Combined with the CATS control difficulties, to improve the accuracy and stability of the CATS in complicated strata, a disturbance observer-based adaptive position control strategy has been proposed. The proposed method presents a nonlinear adaptive controller with adaptation laws to compensate for the unknown time-varying load torque and damping uncertainty. The key feature of the control scheme is that by using the back-stepping technique, we combine the sliding mode control method and disturbance observer method to construct an asymptotically stable controller proven by Lyapunov theory. Simulation experiments are carried out by Simulink and AMESim co-simulation, and the results show the effectiveness of the proposed strategy in the presence of uncertainties.

The contributions of this paper are summarized as follow: first, a disturbance observer-based adaptive position control is successfully designed for CATS of slurry shields. As a result, more robust control performance can be achieved during experiments compared to the PID control. Second, the adaptation laws to compensate for the unknown time-varying load torque and damping uncertainty has shown to improve performance of the CATS. Third, the stability of proposed control is theoretically verified via a Lyapunov function analysis and the error is restricted within a certain range.

## 2. Dynamics model of CATS

**Fig 2:**
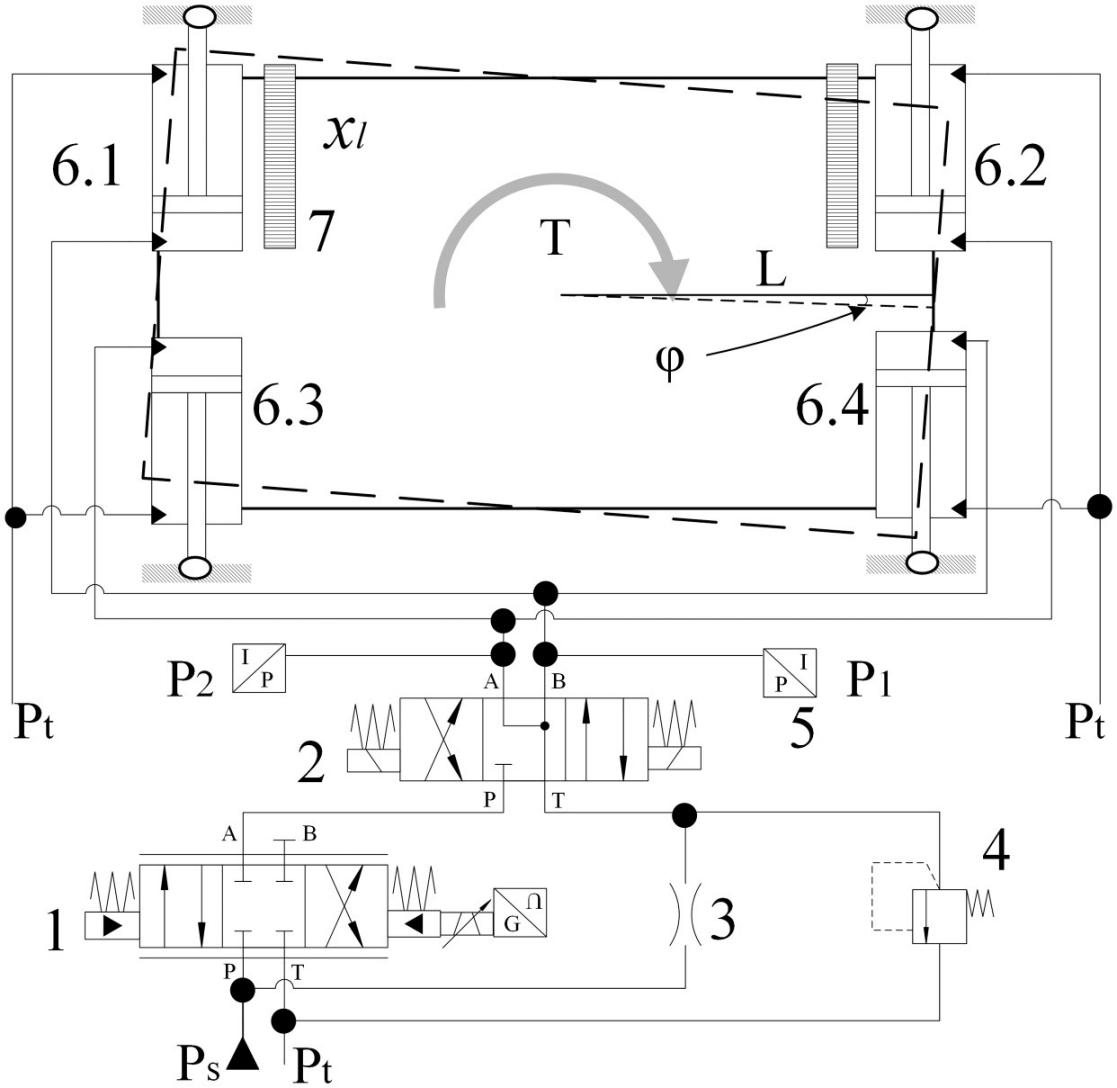
Schematic diagram of the hydraulic system. 1-Servo proportional valve, 2-Directional valve, 3-Fixed orifice, 4-Pressure relief valve, 5-Pressure sensor, 6.1-Torque cylinder I, 6.2 Torque cylinder II, 6.3-Torque cylinder III, 6.4- Torque cylinder IV, 7-Displacement sensor.

The CATS includes four torque cylinders named as torque cylinders I, II, III and IV, as shown in Fig 2. One end of these cylinders is fixed on the drive box, and the other end is hinged to the shield structure. Torque cylinder I and II are equipped with a displacement sensor measuring its rod displacement, respectively. Since the rod-less chambers of the torque cylinder on the diagonal are connected to the same valve port through oil pipes, these cylinders have movement consistency. Their extending or retracting motions depend on the cutterhead sense of rotation. For example, the cutterhead cuts soil counterclockwise, the drive box receives a clockwise torque, torque cylinders I and IV are squeezed and torque cylinders II and III are stretched. In addition, the directional valve sets left way; torque cylinder I and IV are activated and controlled by a servo proportional valve, meanwhile torque cylinder II and III are still passive and maintained with 5 bar hydraulic pressure controlled by the relief valve, to ensure that the passive cylinders are slightly extended and in contact with the shield structure. If the stroke of torque cylinder II is larger than that of torque cylinder I, torque cylinders I and IV will be fed and extended. In contrast, torque cylinders I and IV will be released and retracted. In addition, the directional valve will set the right way if the cutterhead cuts soil clockwise. The torque cylinders can suppress the drive box rolling in real time and smoothly transmit the drive box torque to the shield structure.

For simplicity, take the cutterhead rotating counterclockwise as an example for mathematical model analysis. The directional valve sets left way and ignores the influence of the directional valve on the control system. In addition, the dynamics of the CATS can be given as

$$\begin{array}{l} J\ddot{\varphi }=2(P_{1}-P_{2})AL-(T+B\dot{\varphi })\\ \;\;x_{l}=L\varphi \end{array}$$ (1)

where *J* is the equivalent moment of inertia of the drive box and cutterhead; *φ* is the roll angle of the drive box, *$\dot{\varphi }$* and $\ddot{\varphi }$ are the angular velocity and acceleration, respectively; *P*_1_ and *P*_2_ are the pressures in the active and passive cylinder rod-less chambers, respectively; *A* is the area of the cylinders rod-less side; *L* is the distance from the center of the drive box to the cylinder axis in the horizontal direction; *T* is the cutting anti-torque including some unknown disturbance; *B* is the combined coefficient of damping and viscous friction forces on the bearing, and *x*_*l*_ is the displacement of active torque cylinders. Here, we assume that the roll angle *φ* is very small due to the system working. The relationship between the roll angle *φ* and the active cylinder displacement *x*_*l*_ is linearized, and the drive box angle control can be converted into cylinder displacement control. The following simulation results show that the linearization is reliable in this situation. In addition, the passive torque cylinder displacement is defined as–*x*_*l*_.

Considering the leakage and compressibility of the oil, the pressure dynamic of the active torque cylinder rod-less chamber can be written as

$$Q_{1}=A{\dot{x}}_{l}+C_{t}P_{1}+(V_{0}+Ax_{l})\beta_{e}{}^{-1}{\dot{P}}_{1}$$ (2)

where *Q*_1_ is the fluid flow rate of the active torque cylinder rod-less chamber; ${\dot{x}}_{l}$ is the velocity of active torque cylinders; *C*_t_ is the internal leakage coefficient; *V*_0_ is the initial fluid volume of the active torque cylinder rod-less chamber; *β*_e_ is the bulk modulus of the fluid; and ${\dot{P}}_{1}$ is the derivative of pressure in the active cylinder rod-less chamber.

The fluid flow rate of the servo proportional valve *Q* is related to the spool displacement *x*_*v*_ and given as

$$Q=k_{q}x_{v}[\sigma (x_{v})\sqrt{P_{s}-P_{1}}+\sigma (-x_{v})\sqrt{P_{1}-P_{t}}]$$ (3)

where the function σ (*) is defined as

$$\sigma (*)=\left\{\begin{array}{c} 1\;\;\;\;if\;\;*\;\;\geq 0\\ 0\;\;\;\;if\;\;*\;\;<0 \end{array}\right.$$ (4)

*k*_q_ is the servo proportional valve flow gain coefficient; *P*_s_ is the source pressure, and *P*_t_ is the tank pressure. In addition, port *B* of the servo proportional valve connects two active torque cylinder rod-less chambers, thus *Q* and *Q*_1_ have the following relationship.


$$Q=2\;Q_{1}$$ (5)

Since the dynamics of the servo proportional valve used here are much faster than others, the valve dynamics can be neglected, and the spool displacement is obtained [40] as follows:

$$x_{v}=k_{v}\cdot u$$ (6)

where *k*_*v*_ is a positive constant determined by valve design, and *u* is the control input voltage.

The state variables can be defined as *x* = [*x*_1_, *x*_2_, *x*_3_] ^T^ = [*x*_*l*_, *ẋ*_*l*_, *P*_1_] ^T^. Including (1)–(6), the whole system can be expressed in a state-space form as follows:

$$\begin{array}{l} {\dot{x}}_{1}=x_{2}\\ {\dot{x}}_{2}=2AL^{2}J^{-1}(x_{3}-P_{2})-LJ^{-1}T-BJ^{-1}x_{2}\\ {\dot{x}}_{3}=h{\left(x_{1}\right)}^{-1}[-Ax_{2}-C_{t}x_{3}+\frac{k_{q}k_{v}R(x_{3},u)}{2}u] \end{array}$$ (7)

where

$$\begin{array}{l} R(x_{3},u)=\sigma (x_{v})\sqrt{P_{s}-x_{3}}+\sigma (-x_{v})\sqrt{x_{3}-P_{t}}\\ h(x_{1})=(V_{0}+Ax_{1})\beta_{e}{}^{-1} \end{array}$$ (8)

Under normal conditions, *P*_1_ and *P*_2_ are bounded as 0 ≤ *P*_t_ ≤ *P*_1_ ≤ *P*_s_, *P*_2_ = 5 bar.

## 3. Controller design

### 3.1 Disturbance observer

The disturbance observer we applied here is based on an extended state observer of *x*_2,_ although it is measurable. Thus, an additional degree of freedom for the controller is provided without the actuator acceleration signal. It can be described as

$${\dot{\hat{x}}}_{2}=2AL^{2}J^{-1}(x_{3}-P_{2})-LJ^{-1}\hat{T}-x_{2}J^{-1}\hat{B}+\alpha {\tilde{x}}_{2}$$ (9)

where ${\hat{x}}_{2}$, $\hat{T}$ and $\hat{B}$ are the estimations of *x*_2_, *T* and *B*; ${\dot{\hat{x}}}_{2}$ is the derivative of ${\hat{x}}_{2}$. In addition, ${\tilde{x}}_{2}$ is the estimation error of *x*_2_ and is defined as ${\tilde{x}}_{2}=x_{2}-{\hat{x}}_{2}$, and *α* is a positive feedback gain used to eliminate *x*_2_ error. Including estimation errors $\tilde{T}=T-\hat{T}$ and $\tilde{B}=B-\hat{B}$, the dynamics of ${\tilde{x}}_{2}$ can be expressed as

$${\dot{\tilde{x}}}_{2}=-LJ^{-1}\tilde{T}-x_{2}J^{-1}\tilde{B}-\alpha {\tilde{x}}_{2}$$ (10)

The adaptation law is chosen as

$$\begin{array}{l} \dot{\hat{T}}=Proj_{\hat{T}}(-\beta_{1}LJ^{-1}{\tilde{x}}_{2}+\gamma_{1})\\ \dot{\hat{B}}=-\beta_{2}x_{2}J^{-1}{\tilde{x}}_{2}+\gamma_{2} \end{array}$$ (11)

where *β*_1_ and *β*_2_ are positive constants; *γ*_1_ and *γ*_2_ are additional corrector parts designed to ensure the stability of the whole system; $\dot{\hat{T}}$ and $\dot{\hat{B}}$ are the derivative of $\hat{T}$ and $\hat{B}$; and $Proj_{\hat{d}}(\cdot )$ is defined as

$$Proj_{\hat{T}}(•)=\left\{\begin{array}{l} 0\\ 0\\ • \end{array}\right.\begin{array}{cc} & \begin{array}{l} \begin{array}{cccc} if & \hat{T}=T_{\text{max}} & and & •>0 \end{array}\\ \begin{array}{cccc} if & \hat{T}=T_{\text{min}} & and & •<0 \end{array}\\ \begin{array}{cccc} & otherwise. & & \end{array} \end{array} \end{array}$$ (12)

And it can be found that $\hat{T}\in \left[T_{\text{min}},T_{\text{max}}\right]$.

Define the following Lyapunov function *V*_1_ as

$$V_{1}=\frac{1}{2}{\tilde{x}}_{2}^{2}+\frac{1}{2}\beta_{1}^{-1}{\tilde{T}}^{2}+\frac{1}{2}\beta_{2}^{-1}{\tilde{B}}^{2}$$ (13)

The time derivative of *V*_1_ is given by

$${\dot{V}}_{1}=-\alpha {\tilde{x}}_{2}^{2}-\beta_{1}^{-1}\tilde{T}\gamma_{1}-\beta_{2}^{-1}\tilde{B}\gamma_{2}$$ (14)

*Remark 1*: Since there is no more detailed information about the derivative of the load torque *T* and friction coefficient *B*, it is reasonable to assume that *T* and *B* vary slowly relative to the observer dynamics [41]; then, $\dot{T}\approx 0$ and $\dot{B}\approx 0$. The following simulation shows that the proposed controller is reliable in this situation.

### 3.2 Nonlinear position control

The nonlinear controller consists of a tracking control outer loop and a pressure control inner loop which provides the hydraulic actuator the characteristics of a force generator. Furthermore, sliding mode control is adopted to compensate for estimation errors. By using the recursive back-stepping technique, the controller is designed as follows.

Step 1: The goal of the step is to design a desired load force for the tracking control outer loop. This controller must be robust to estimation errors. Here, we define the tracking error as ${\tilde{x}}_{1}=x_{1}-x_{d}$, and *x*_*d*_ = 0. Then, a sliding mode surface *s* is defined as

$$s={\dot{\tilde{x}}}_{1}+\lambda {\tilde{x}}_{1}$$ (15)

where *λ* is a positive constant. Making the tracking error small or converging to zero is the same as making *s* small or converging to zero, so the next process is to make *s* as small as possible.

From (15), the time derivative of *s* along system (7) is expressed as

$$\dot{s}=2AL^{2}J^{-1}(x_{3}-P_{2})-LJ^{-1}T-BJ^{-1}x_{2}+\lambda x_{2}$$ (16)

In the pressure control inner loop, design a virtual controller *y*_3_ for *x*_3_ as

$$y_{3}=P_{2}\text{+}[LJ^{-1}\hat{T}+x_{2}J^{-1}\hat{B}-\lambda x_{2}-\eta_{1}s]J/(2AL^{2})$$ (17)

where *η*_1_ is a positive constant used to eliminate pressure tracking error. Here, we define the tracking error of the pressure control inner loop as *z*_3_ = *x*3 − *y*_3_. Combining (16) and (17), we can obtain

$$\dot{s}=-LJ^{-1}\tilde{T}-x_{2}J^{-1}\tilde{B}-\eta_{1}s+2AL^{2}J^{-1}z_{3}$$ (18)

Define the following Lyapunov function *V*_2_ as

$$V_{2}=V_{1}+\frac{1}{2}\beta_{3}^{-1}s^{2}$$ (19)

where *β*_3_ is a positive constant.

The time derivative of *V*_2_ is given by

$${\dot{V}}_{2}=-\alpha {\tilde{x}}_{2}^{2}-(\beta_{1}^{-1}\gamma_{1}+Ls\beta_{3}^{-1}J^{-1})\tilde{T}-(\beta_{2}^{-1}\gamma_{2}+x_{2}s\beta_{3}^{-1}J^{-1})\tilde{B}-\beta_{3}^{-1}\eta_{1}s^{2}+\beta_{3}^{-1}2AL^{2}J^{-1}z_{3}s$$ (20)

Then, the additional corrector parts mentioned before are chosen as

$$\begin{array}{l} \gamma_{1}=-Ls\beta_{3}^{-1}J^{-1}\beta_{1}\\ \gamma_{2}=-x_{2}s\beta_{3}^{-1}J^{-1}\beta_{2} \end{array}$$ (21)

Substitute (21) into (20), and we can get

$${\dot{V}}_{2}=-\alpha {\tilde{x}}_{2}^{2}-\beta_{3}^{-1}\eta_{1}s^{2}+2AL^{2}J^{-1}z_{3}s\beta_{3}^{-1}$$ (22)

Step 2: In step 1, we have designed a virtual control law *y*_3_ which is the command input of the inner load pressure control loop. In this step, an actual control law for *u* is determined.

From (7), the time derivative of *z*_3_ is given by

$${\dot{\text{z}}}_{\text{3}}=h{\left(x_{1}\right)}^{-1}[-Ax_{2}-C_{t}x_{3}+\frac{k_{q}k_{v}R(x_{3},u)}{2}u]-{\dot{y}}_{3}$$ (23)

And the actual control *u* is designed as

$$\begin{array}{l} u=\frac{2u_{x}}{k_{q}k_{v}R(x_{3},u)}\\ u_{x}=Ax_{2}+C_{t}x_{3}+h(x_{1})({\dot{y}}_{3}-\eta_{2}z_{3}-\beta_{3}^{-1}\beta_{4}2AL^{2}J^{-1}s) \end{array}$$ (24)

where *η*_2_ and *β*_4_ are both positive constants.

*Remark 2*: Note that (24) includes the control input *u* on both sides of the equation, so (24) cannot be calculated directly. However, the control input *u* on the right side is only used for *R*(·), since *R*(·) is always greater than zero, the sign of *u* is determined by *u*_*x*_.

Substitute (24) into (23), and we can get

$${\dot{\text{z}}}_{\text{3}}=-\eta_{2}z_{3}-\beta_{3}^{-1}\beta_{4}2AL^{2}J^{-1}s$$ (25)

Define the following Lyapunov function *V*_3_ as

$$V_{3}=V_{2}+\frac{1}{2}\beta_{4}^{-1}z_{3}^{2}\;\;$$ (26)

From (26), the time derivative of *V*_3_ is given by

$${\dot{V}}_{3}=-\alpha {\tilde{x}}_{2}^{2}-\beta_{3}^{-1}\eta_{1}s^{2}-\beta_{4}^{-1}\eta_{2}z_{3}^{2}$$ (27)

It is obvious that ${\dot{V}}_{3}\leq 0$. The stability of the closed-loop system is guaranteed by Lyapunov theory, and all system signals are bounded under closed-loop control.

*Theorem 1*: Lyapunov’s second method for stability makes use of a Lyapunov function *V*(x) which has an analogy to the potential function of classical dynamics. It is introduced as follows for a system $\dot{x}=f(x)$ having a point of equilibrium at *x* = 0. Consider a function *V*: *R*^*n*^ → *R* such that

$$\begin{array}{l} V(x)=0\;if\;and\;only\;if\;x=0;\\ V(x)>0\;if\;and\;only\;if\;x\neq 0;\\ \dot{V}(x)=\frac{d}{dt}V(x)=\nabla V\cdot f(x)=\sum_{i=1}^{n}\frac{\partial V}{\partial x_{i}}f_{i}(x)\leq 0\;for\;all\;values\;of\;x\neq 0. \end{array}$$

Then *V*(x) is called a Lyapunov function and the system is stable in the sense of Lyapunov.

**Fig 3:**
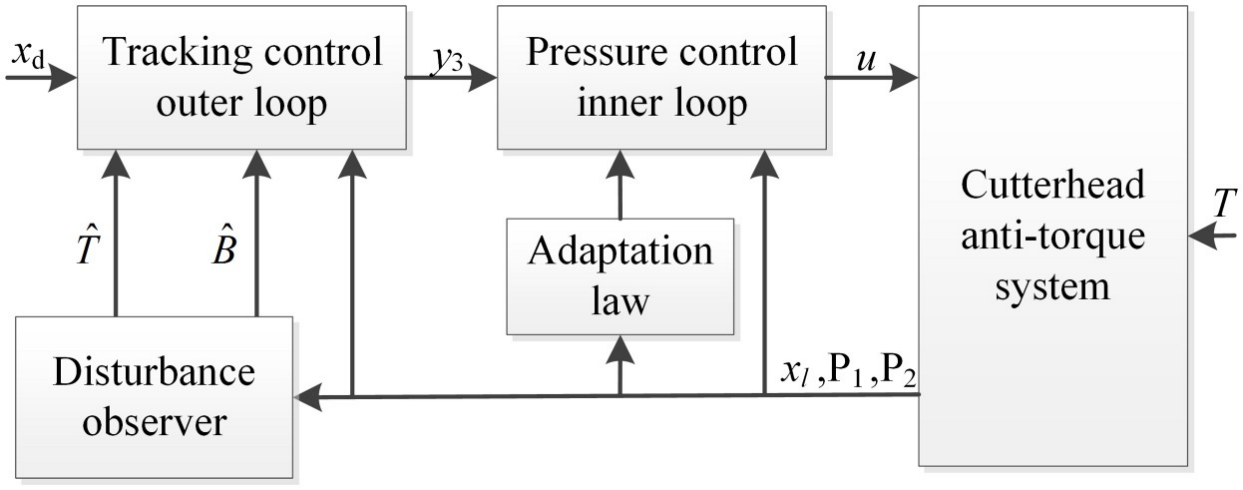
Block diagram of the controller structure.

The block diagram of the adaptive controller structure is shown in Fig 3.

## 4. Simulation results

**Fig 4:**
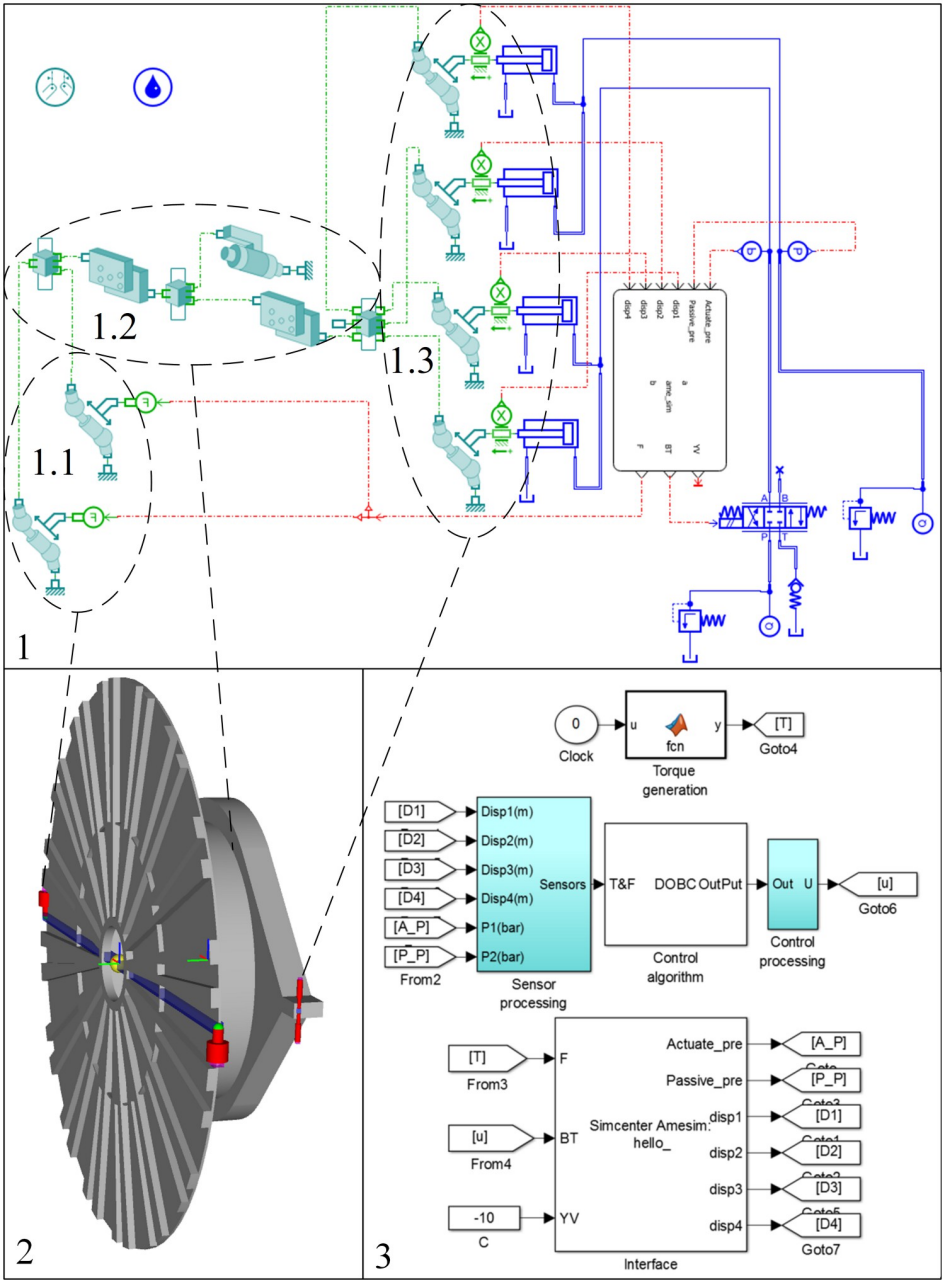
Virtual test rig of the CATS. 1-AMESim simulation model of the CATS, 1.1-Load cylinder, 1.2-Mechanism, 1.3-Torque cylinder, 2-Virtual 3D structure built by AMESim, 3-MATLAB control algorithm.

To test the proposed disturbance observer-based adaptive position control, simulations are performed using the MATLAB and AMESim co-simulation method. The virtual test rig shown in Fig 4 is mainly composed of 4 torque cylinders, 2 load cylinders and their matching electrohydraulic system. And dimensions of the torque cylinders are 600mm/520mm/400mm. The servo proportional valve used is the 4WRPH6C4B40L-type valve made by Rexroth, and the control input is [–10,10] V. The mechanism structure and size are close to those of a fifteen-meter-diameter slurry shield numbered S-1046 produced by Herrenknecht Ag. Torque cylinders can be controlled by one servo proportional valve, and then the attitude of the drive box can be adjusted precisely. In addition, the force of the load cylinders can be set to simulate the load torque and cause a rotation of the drive box.

To verify the performance of the adaptive position controller, the external load force F is set as shown below:

$$F=\left\{\begin{array}{cc} \;\;\;\;(2-2\text{cos}(\pi t))*10^{5}\;\;\;\;\;\;\;N & \;\;\;\;if\;t\;\;<1s\;\;\;\;\\ \;\;\;\;(7-3\text{sin}(0.5\pi t))*10^{5}\;\;N & else \end{array}\right.$$ (28)

In addition, disturbance is unknown, and *x*_*d*_ is 0. The parameters of CATS and controller are chosen according to Tables 1 and 2. And the proposed control parameters are well tuned.

**Table 1:** The parameters of CATS.

Parameter	Description	Value
*J*	Moment of inertia of the drive box and cutterhead	2.45*10^6^ kg·m^2^
*A*	Cylinder chamber area	0.283 m^2^
*V*_*0*_	Cylinder chamber initial volume	5.66 * 10^−2^ m^3^
*L*	Distance from the center of the drive box to the cylinder axis	5.00 m
*P*_*s*_	Source pressure	2.50*10^7^ Pa
*P*_*t*_	Tank pressure	0 Pa
*K*_*q*_ *·K*_*v*_	Valve flow gain coefficient	1.43 * 10^−7^ m^3^/(s · V · $\sqrt{Pa}$)
*β*_*e*_	Oil bulk modulus	1.00*10^9^ Pa
*C*_*t*_	Cylinder leakage coefficient	1.00 * 10^−14^ m^3^/(s·Pa)

**Table 2:** The parameters of proposed controller.

Parameter	Value	Parameter	Value
*β*_*1*_	1.25 * 10^12^	*η*_*2*_	12
*β*_*2*_	1.25 * 10^12^	*α*	2000
*β*_*3*_	0.001	*λ*	250
*β*_*4*_	0.01	$\hat{T}(0)$	0
*η*_*1*_	120	$\hat{B}(0)$	0

Traditional PID control is compared with the proposed control in this paper. And the input control *u* of PID control is given by

$$u=2000(x_{d}-x_{1})+500\int_{0}^{t}(x_{d}-x_{1})d_{t}+100\frac{d(x_{d}-x_{1})}{d_{t}}$$ (29)

The PID control algorithm parameters are well tuned and used in the whole process.

**Fig 5:**
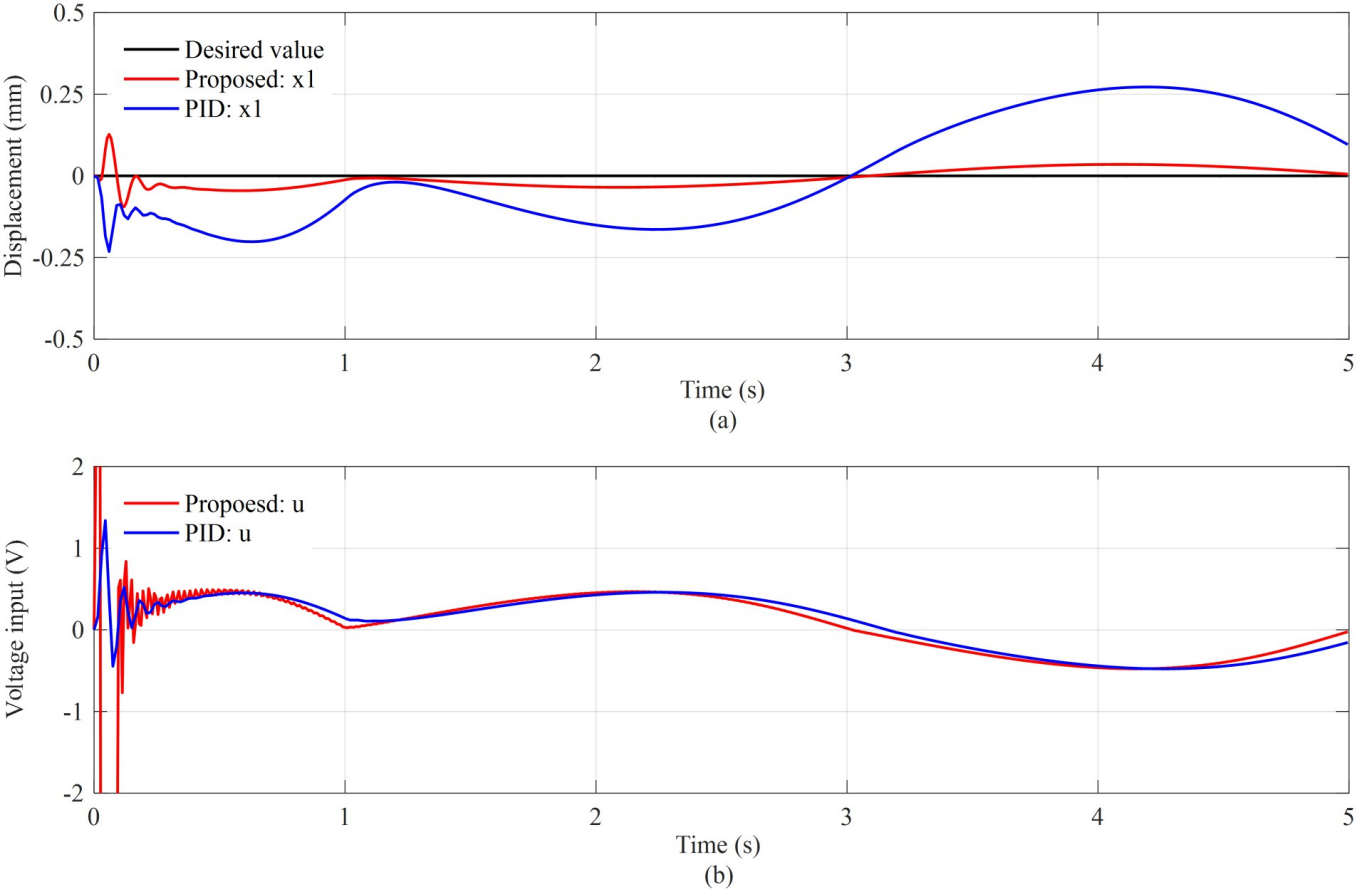
Tracking performance (a) and control input voltages (b) of the two controller methods.

The desired displacement of the torque cylinders is shown as a black line in Fig 5a. The two controllers drive each torque cylinder to track its desired displacement as closely as possible to prevent the drive box from rotation. The red line and blue line present the tracking performance of the proposed method and PID method, respectively. The proposed controller performs better than the PID controller in terms of position tracking error. The tracking error of the PID method is more than twice that of the proposed method which is bounded within ±0.13 mm. At the beginning, the cutterhead anti-torque system with the proposed controller cannot follow the desired trajectory smoothly due to the difference between the initial and desired system states. However, after 0.3 seconds of adjustment, the displacement of the torque cylinders is appropriately controlled and bounded within ±0.05 mm. It can be considered that the torque cylinders are well controlled during the entire stroke process. The control input voltages of servo proportional valves are shown in Fig 5b. Without considering the adjusted state, the control input values are reasonable, laying the foundation for smooth responses of the torque cylinders.

**Fig 6:**
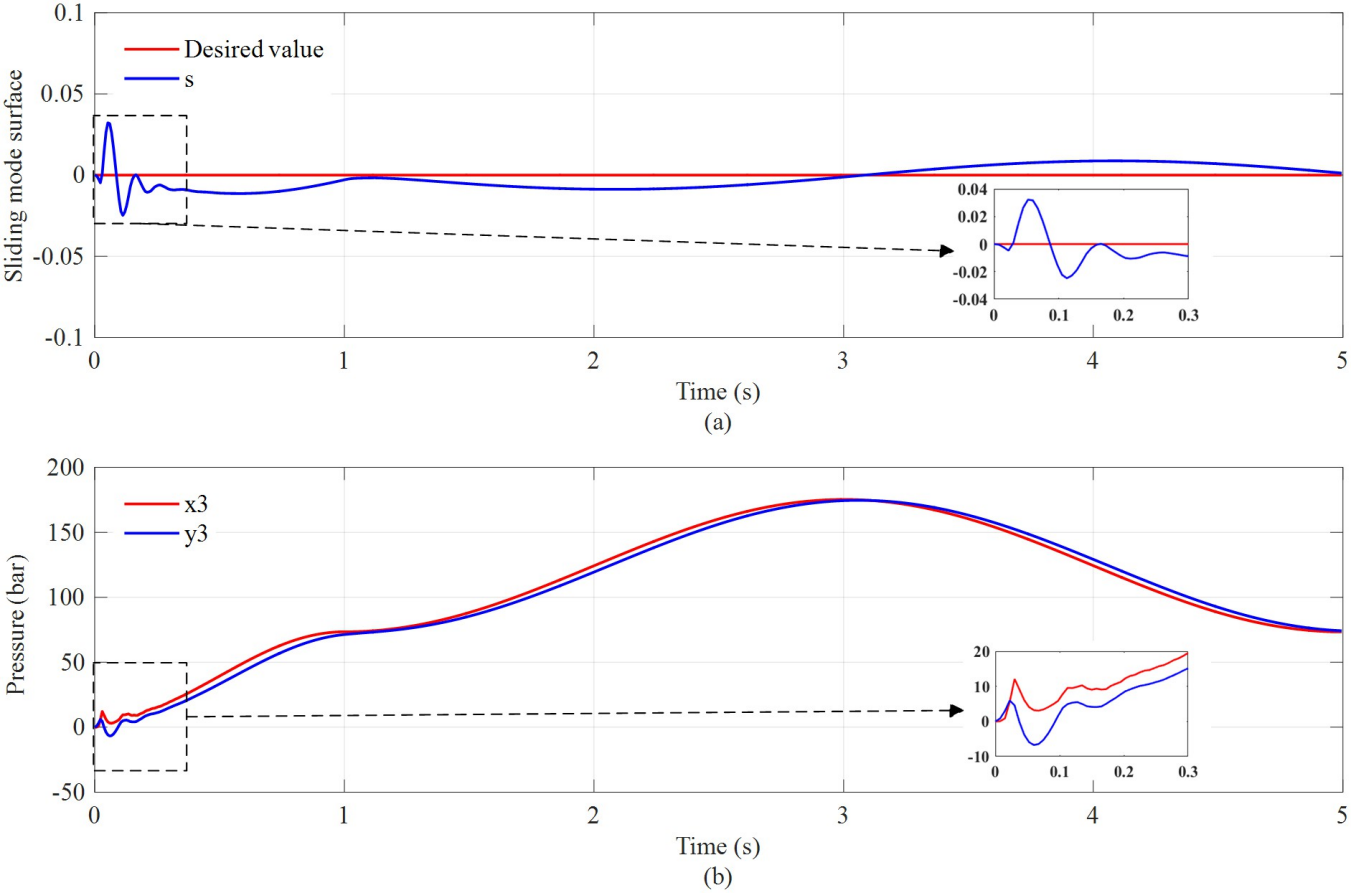
Sliding mode surface (a) and pressure in the rodless chamber of the active torque cylinder (b).

Moreover, the error of sliding surface *s* shown in Fig 6a is also bounded within ±0.04, and after its adjustment, the error is constrained within ±0.01, indicating that, similar to the displacement, the velocity of torque cylinders is well controlled, and their tracking errors are small. This shows that the tracking control outer loop performs well. Fig 6b shows that the nominal controller *y*_3_ tracks the desired *x*_3_ value, and the maximum error that appears at the beginning is less than 10 bar. After its adjustment, the error is bounded within ±5 bar. Thus, it can be considered that the pressure control inner loop performs well.

**Fig 7:**
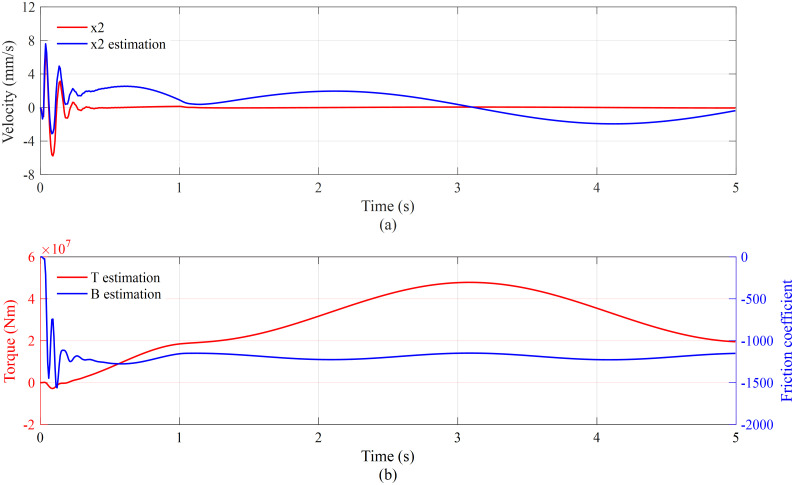
Velocity estimation (a) and torque and friction coefficient estimation (b).

Fig 7a shows the velocity estimation of torque cylinders based on the disturbance observer. The estimation of velocity is relatively close to the actual velocity. In addition, the estimation error is bounded within ±3 mm/s. Fig 7b presents the torque estimation and the friction coefficient estimation based on the adaptation law. The trend of torque estimation is similar to that of load force, and the estimation of the combined friction coefficient is stable at approximately -1100. Since the displacement and pressure track well, it can be considered that the torque and the friction coefficient are estimated well.

Above all, the simulation results prove strongly that the proposed controller has good performance and great control accuracy in the presence of uncertainties.

## 5. Conclusion

In this work, disturbance observer-based adaptive position control for a cutterhead anti-torque system is proposed. The proposed method presents a nonlinear adaptive controller with adaptation laws to compensate for the unknown time-varying load torque and damping uncertainty. By using the back-stepping technique, an asymptotically stable controller proven by Lyapunov theory is constructed based on the disturbance observer method and sliding mode control. In addition, the validity of the proposed controller is verified by MATLAB and AMESim co- simulation. The simulation results show good performance for the tracking task in the presence of uncertainties compared with the traditional PID strategy. Together, the data support targeting disturbance observer-based adaptive position control as a potential control strategy for cutterhead anti-torque systems.

## Supporting information

S1 Data (ZIP)
